# The Game of the Right Hand against the Left

**Published:** 1878-07

**Authors:** P. Harold Hayes


### THE GAME OF THE RIGHT HAND AGAINST THE LEFT.

P. HAROLD HAYES, M. D.

A carriage drew up before my door on a bright June day. A young lady stepped out and came briskly up the walk, and as she drew near, I recognized in her a former patient. She had been sent from India to this country, some years before, for an education, and had lost her health, at Mt. Holyoke Seminary. She asked me to go down to the carriage, wherein, as I approached, I saw an improvised bed, and when a little nearer my guide introduced me to its occupant by saying, "My sister, doctor, another victim of Mt. Holyoke." And now I never see a "*victim*" of confinement, and overworked brains from a young ladies' boarding school, that the words of this introduction, "another victim of Mt. Holyoke," do not flash through my mind.

Not long before this I had been invited with my family to the performance of a *Cantata* at Madame C—'s Seminary, and on the following morning my daughter said, "Father, how did you like the *Cantata* last night?" and I replied, the singing was excellent, the parts were well-sustained, and the white dresses, gaily festooned with flowers, the wreaths and the garlands, and above all, the bright faces of the girls, lent such a charm, as girls only can lend to any scene in life. I could not help observing, however, that though there was no lack in brains among them, there was a great lack as to bodies. Some of them were thin, nearly all were wanting in the roundness and figure, which more muscular development would give. Very few of that troop of girls will ever fill out the full outline, and fair proportions of a normal womanhood.

How can they? They have almost none of those free exercises in which the element of play and abandon enter at will. In this way only can the animus of a free spirit, penetrate the limbs and carry with it the blood, tingling to every part of the body. Without such exercises they cannot have bodily *vigor* and without this, they cannot bear fatigue, or heat, or cold, or the most ordinary and inevitable exposures. Exercise, we say, develops and strengthens every part of the body, but this is true only in a limited sense, as to the brain. When this is exercised so constantly and without the balancing and bracing effect of bodily exercise, its activity and its impressibility are intensified, but it neither grows nor is it strengthened. There cannot be growth and strength in the brain without abundant and rich blood, and there cannot be abundant blood-making without abundant exercise.

The muscles are fully one-half the weight of a strong girl, and actually receive two-thirds of all her blood. The whole normal amount of blood in the body is about one-seventh part of its weight, or fifteen pounds in a girl weighing a hundred and five pounds; but this proportion will fail in every young miss I saw last evening, and some of them have no more than two-thirds of the normal proportion. They come short in blood by so much as they lack in muscle, and by so much as they come short in both blood *and* muscle, will they fail in permanent brain power.

In your ranks last night I saw many good looking girls; nature thought to make them beautiful. She cannot do it; they are shut up in doors, they want muscle, figure, blood, color; they have indeed a flush, a fever, when excited, but they will rise in the morning, pale and languid. Madame De Stael is said to have declared she would give half her knowledge for personal charms. I supposed girls knew by a sort of natural sagacity, the value of beauty; and if they do, they ought, also, to know that without *health*, beauty is impossible. Superadded to our common human nature, has every girl her woman nature, which through all these years is undergoing the most positive, though half unconscious evolution, and her imagination takes hold on some, perhaps purely ideal, counterpart of herself, on whose arm she shall some day lean, and who shall give her the word and "the one look" no other man does or dare. Blessed be this God-given aspiration, never yet breathed in words, but how can it come to crownings and rejoicings through weakness? Does not nature and history everywhere teach the broad lesson, that to be weak is to be miserable?

Remember muscle stands for blood, nutrition, color, power, and from power comes courage, self-possession, and endurance. Some of these girls will soon have to consult the doctor, and he will discover that their malady has been slowly wrought into the grain and fibre of their existence, and the cure, if ever it be radical, must be long deferred. Even now some of them are "very nervous," have headaches, imperfect sleep. It is a game of the right hand against the left, a war among the members—brains against muscle, mind against body—and body will win, not by strength, but by the might of *weakness*; strange paradox! "mighty weak!"

It is a great misfortune for girls to be entirely separated from the employments of home, while acquiring knowledge from books in schools. Girls should elevate and honor by doing them, all parts of the work belonging to the keeping and adorning of a home, in house, garden and grounds, and in the rearing and training of pet animals. There is no substitute for these employments, on the large scale, as health-inspiring exercises. They can be as truly *artistes* in the details of home-life, as in their dress or with their pencil. How many are "sand blind" to these opportunities for health and enjoyment, just at their right hand!

But, Father, you know they have their *regular* gymnastics in the Seminary. Yes, and the regularity is a part of the vice of them. They dull and tire by monotony. Some of them are essentially unnatural, and their very character of method and drill, makes them discipline, rather than recreation. We cannot long beat the air by rule, and find the virtue of exercise in it, no more than we can love, or laugh, or frolic by rule. Do you not remember the two young ladies we saw at B—? They had just graduated at a seminary of note, and so important did this event seem to them, that all recent and even long previous transpirings were dated from this epoch; so long before, or so long after they *graduated* at P——. They were well-trained in books, music, and almost to affectation in the proprieties. They were girls of some true pride, and saw, as I think, something of the absurdity of going fresh with academic honors, into a sanitarium for invalids. The mother was strong and able to lead about and be servant to her tender daughters. These were thin, dyspeptic, bloodless, their fate (?) *invalidism. Cui bono? Doing*, not thinking only, makes character. Here were learning and accomplishments, and in the getting, disuse and loss of the power to enjoy them. They did not seem to have any vivacity; nature was schooled out of them. They went about exercise as though it were something ecclesiastical, judicial, or expiatory. They had no natural sense of fatigue, therefore no sound and refreshing sleep. They had no genuine sense of *hunger*, the true seasoning for our food. They came down in the morning with the *bouche pateuse*, or clammy mouth, and would look wistfully around the table for some sharp sauce to quicken appetite, and end by saying, "Can't eat—everything tastes so horrid!" Can that be called education in any true sense which brings a young woman to this? We would like to answer as a Frenchman would, *ordinairement jamais*.

—Send for a beautifully bound copy of the Bistoury, containing the numbers for past three years, and fully indexed. Price $2.50.

